# Differential diagnosis between LQT1 and LQT2 by QT/RR relationships using 24‐hour Holter monitoring: A multicenter cross‐sectional study

**DOI:** 10.1111/anec.12878

**Published:** 2021-07-10

**Authors:** Kenji Yodogawa, Takeshi Aiba, Naotaka Sumitomo, Teppei Yamamoto, Hiroshige Murata, Yu‐ki Iwasaki, Yoshihiro Kokubo, Wataru Shimizu

**Affiliations:** ^1^ Department of Cardiovascular Medicine Nippon Medical School Tokyo Japan; ^2^ Department of Advanced Arrhythmia and Translational Medical Science National Cerebral and Cardiovascular Center Osaka Japan; ^3^ Department of Pediatric Cardiology Saitama Medical University International Medical Center Saitama Japan; ^4^ Department of Preventive Cardiology National Cerebral and Cardiovascular Center Suita Japan

**Keywords:** ECG, Holter monitoring, long QT syndrome, QQ/RR relationships

## Abstract

**Background:**

The clinical course and therapeutic strategies in the congenital long QT syndrome (LQTS) are genotype‐specific. However, accurate estimation of LQTS genotype is often difficult from the standard 12‐lead ECG.

**Objectives:**

This study aims to evaluate the utility of QT/RR slope analysis by the 24‐hour Holter monitoring for differential diagnosis of LQTS genotype between LQT1 and LQT2.

**Methods:**

This cross‐sectional study enrolled 54 genetically identified LQTS patients (29 LQT1 and 25 LQT2) recruited from three medical institutions. The QT‐apex (QTa) interval and the QT‐end (QTe) interval at each 15‐second were plotted against the RR intervals, and the linear regression (QTa/RR and QTe/RR slopes, respectively) was calculated from the entire 24‐hour and separately during the day or night‐time periods of the Holter recordings.

**Results:**

The QTe/RR and QTa/RR slopes at the entire 24‐hour were significantly steeper in LQT2 compared to those in LQT1 patients (0.262 ± 0.063 vs. 0.204 ± 0.055, *p* = .0007; 0.233 ± 0.052 vs. 0.181 ± 0.040, *p* = .0002, respectively). The QTe interval was significantly longer, and QTe/RR and QTa/RR slopes at daytime were significantly steeper in LQT2 than in LQT1 patients. The receiver operating curve analysis revealed that the QTa/RR slope of 0.211 at the entire 24‐hour Holter was the best cutoff value for differential diagnosis between LQT1 and LQT2 (sensitivity: 80.0%, specificity: 75.0%, and area under curve: 0.804 [95%CI = 0.68–0.93]).

**Conclusion:**

The continuous 24‐hour QT/RR analysis using the Holter monitoring may be useful to predict the genotype of congenital LQTS, particularly for LQT1 and LQT2.

## 1 INTRODUCTION

Congenital long QT syndrome (LQTS) is a hereditary disorder characterized by prolonged QT interval and fatal ventricular arrhythmias (Schwartz et al., 1975; Shimizu, 2005). The clinical course and the treatment consideration in the congenital LQTS are genotype‐specific. The most frequent types of LQTS are LQT1 and LQT2, caused by mutations in genes of the potassium channels. Cardiac events are often associated with a sympathetic response by physical stress in LQT1 patients, and beta‐blockers are more effective than those in LQT2 patients (Moss et al., 2007; Shimizu et al., 2009). Therefore, the differential diagnosis between LQT1 and LQT2 is important but can be difficult with standard 12‐lead ECG. The QT–RR relationship using Holter ECG recordings is a novel method for evaluating QT adaptation to the heart rate change, and it has been reported to be useful for detecting LQTS. Patients with LQTS showed an abnormal prolongation of the QT intervals at lower heart rate, resulting in a steeper QT/RR slope than in controls (Merri et al., 1992; Neyroud et al., 1998). Furthermore, previous studies suggested that the heart rate dependence of QT interval was steeper in LQT2 than in LQT1, and QT intervals at slower heart rate were longer in LQT2 patients than those in LQT1 patients (Nemec et al., 2004; Viitasalo et al., 2002). Therefore, QT/RR relationship obtained from Holter monitoring may be useful for differential diagnosis between LQT1 and LQT2.

In the present study, we aimed to further evaluate the utility of QT/RR slope by 24‐hour Holter monitoring by examining that separately at daytime and at nighttime for differential diagnosis between LQT1 and LQT2.

## 2 METHODS

This prospective cross‐sectional study included 29 LQT1 patients genetically identified, and 25 LQT2 patients (mean age 23.4±14.9 years, seven males) recruited from three medical institutions from April 2014 to March 2019.

### 2.1 Genetic studies

In the present study, all patients were already genetically diagnosed with LQT1 or LQT2 by extracting genomic DNA from the leukocytes, then using a combination of polymerase chain reaction, either denaturing high‐performance liquid chromatography or single‐stranded conformation polymorphism and DNA sequencing.

### 2.2 Standard 12‐lead ECGs

We used a standard 12‐lead ECG tracing at 25‐mm/s paper speed and 10‐mm/mV amplitude. Their standard 12‐lead ECG was without any suspicious abnormalities (e.g., signs of ventricular hypertrophy, intraventricular conduction disturbances) except QT prolongation.

### 2.3 Holter ECG

**FIGURE 1:**
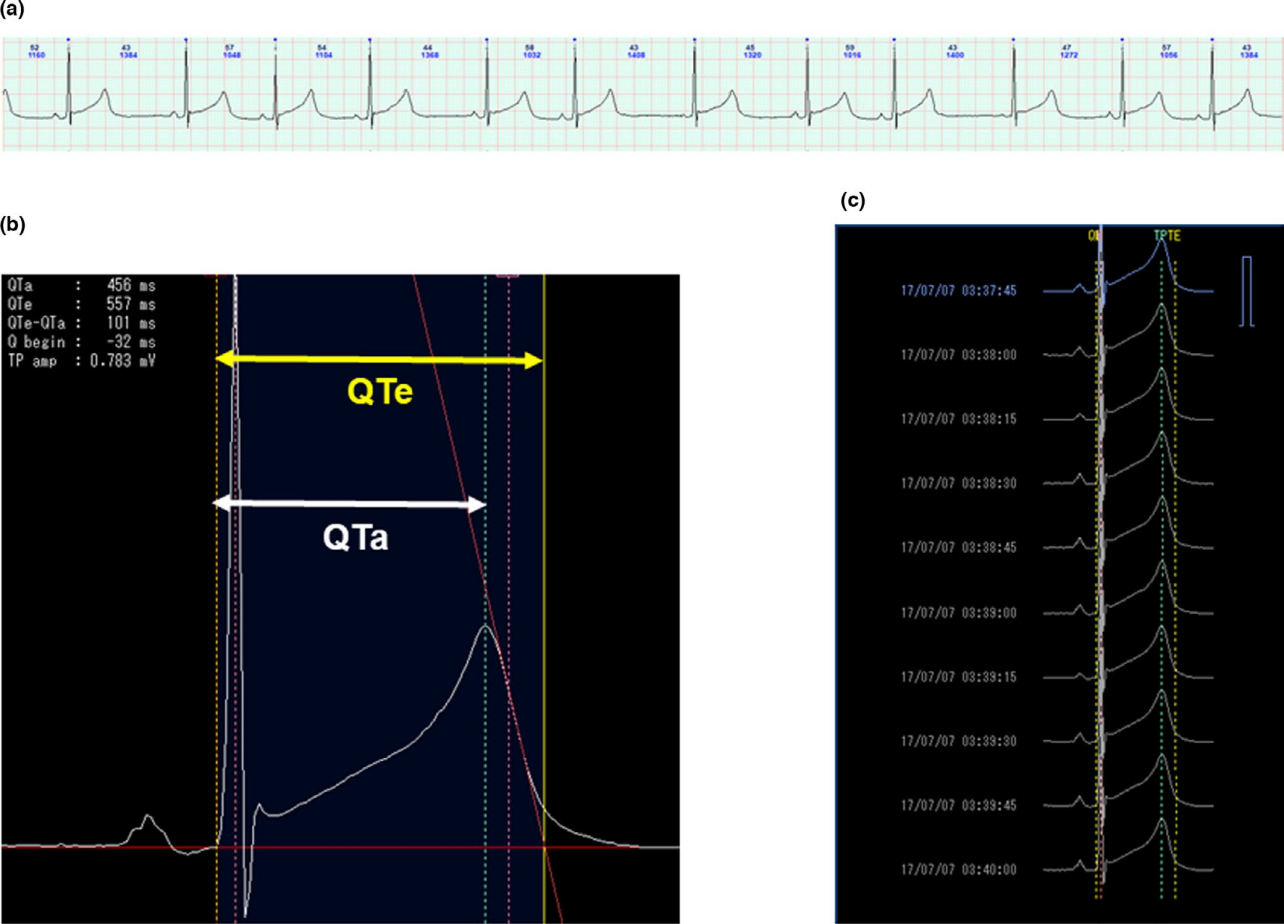
QT measurement. (a) Consecutive sinus beats every 15 s were selected and averaged. (b) Signal averaged waveform. The QTe interval was defined as the time between the QRS onset and the point at which the isoelectric line intersected a line tangent to the maximal (or minimal) downslope of the positive (or negative) T wave. The QTa interval was defined as the time between the QRS onset and the T‐wave's apex (or nadir). (c) This process was repeated every 15 s

A digital ECG recording device (Kenz Cardy 303 pico+; SUZUKEN Co., Ltd.) with a sampling rate of 125Hz was used with an automatic measurement system (Kenz Cardy Analyzer 05^®^; SUZUKEN Co., Ltd.). Consecutive sinus beats every 15 s were averaged, and each parameter was measured. The rate‐corrected QT interval (QTc interval) was determined according to the Bazett formula. The QTe interval was defined as the time between the QRS onset and the point at which the isoelectric line intersected a line tangent to the maximal (or minimal) downslope of the positive (or negative) T wave. The QTa interval was defined as the time between the QRS onset and the apex (or nadir) of the T wave (Figure 1). The linear regression slopes of the QTa interval and the QTe interval plotted against RR intervals (QTa/RR and QTe/RR slopes, respectively) were calculated by the least‐squares method. The QTe‐QTa interval was defined as the time between the QT apex and the QT end and was also plotted against RR intervals (QTe‐QTa/RR slope). These data were compared between a non‐sleep period (daytime) and an actual sleep period (nighttime).

### 2.4 Statistical analysis

Measurements are presented as mean value ± SD. Comparisons of measurements between two groups were analyzed by Mann–Whitney U test. Fisher's exact test was used for discrete variables. Receiver‐operator characteristics (ROC) curves were used to optimize each parameter's cutoff value for differentiation between LQT1 and LQT2.

A *p* value < .05 was considered significant. Statistical calculations were performed with SPSS version 20 software (IBM Inc.).

## 3 RESULTS

### 3.1 Clinical characteristics

The clinical characteristics of both groups are shown in Table 1. No significant differences were found in age, sex, use of beta‐blockers, and history of syncope.

**TABLE 1:** Comparison of each parameter between LQT1 and LQT2 patients

	LQT1 (*n* = 29)	LQT2 (*n* = 25)	*p* value
Age, years	21.6 ± 15.0	25.6 ± 14.8	.328
Male, *n*	3	4	.833
Beta‐blockers	18	19	.421
Syncope	19	19	.552
QTa (average), ms	379.6 ± 34.8	398.1 ± 34.5	.488
QTe (average), ms	447.1 ± 44.8	472.0 ± 40.6	.037
QTec (average), ms	476.8 ± 32.5	487.2 ± 34.7	.269
QTa/RR (whole day)	0.181 ± 0.040	0.233 ± 0.052	.0002
QTa/RR (daytime)	0.153 ± 0.050	0.190 ± 0.048	.008
QTa/RR (nighttime)	0.158 ± 0.048	0.150 ± 0.047	.514
QTe/RR (whole day)	0.204 ± 0.055	0.262 ± 0.063	.0007
QTe/RR (daytime)	0.158 ± 0.066	0.197 ± 0.057	.024
QTe/RR (nighttime)	0.179 ± 0.064	0.168 ± 0.058	.490
QTe‐QTa/RR (whole day)	0.023 ± 0.028	0.029 ± 0.032	.456
QTe‐QTa/RR (daytime)	0.005 ± 0.028	0.007 ± 0.031	.802
QTe‐QTa/RR (nighttime)	0.021 ± 0.026	0.018 ± 0.030	.674

### 3.2 Holter analysis

**FIGURE 2:**
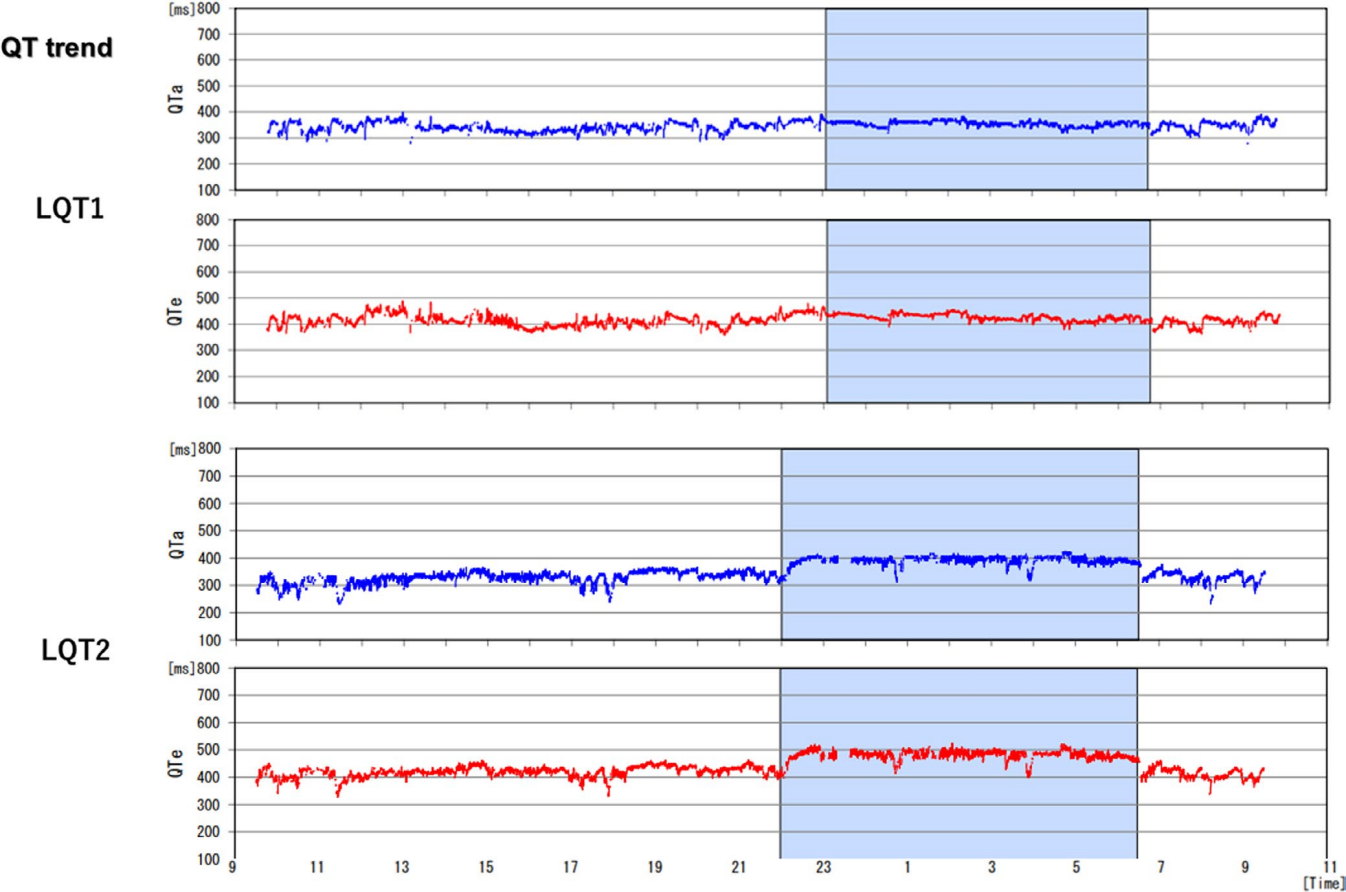
Representative the trend of QT interval along with the 24‐hour study in each group. QT trend graph of the LQT2 showed that QT prolongation was more prominent in the nighttime

**FIGURE 3:**
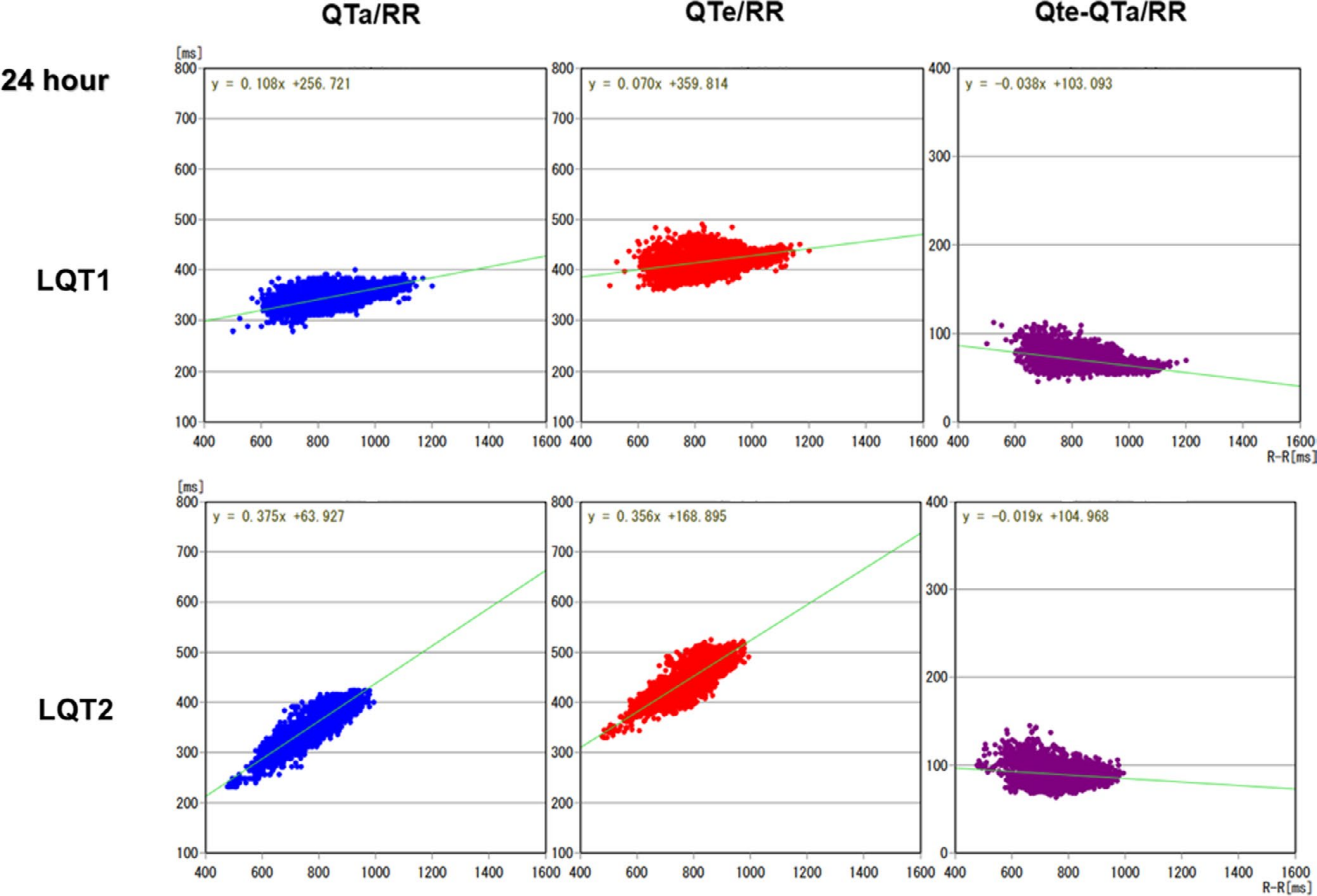
Representative QTa/RR, QTe/RR, and QTe‐QTa/RR slopes from entire 24‐hour Holter recordings in the LQT1 and LQT2 patients. QTa/RR and QTe/RR slopes were steeper in the LQT2 patient than that of the LQT1 patient

**FIGURE 4:**
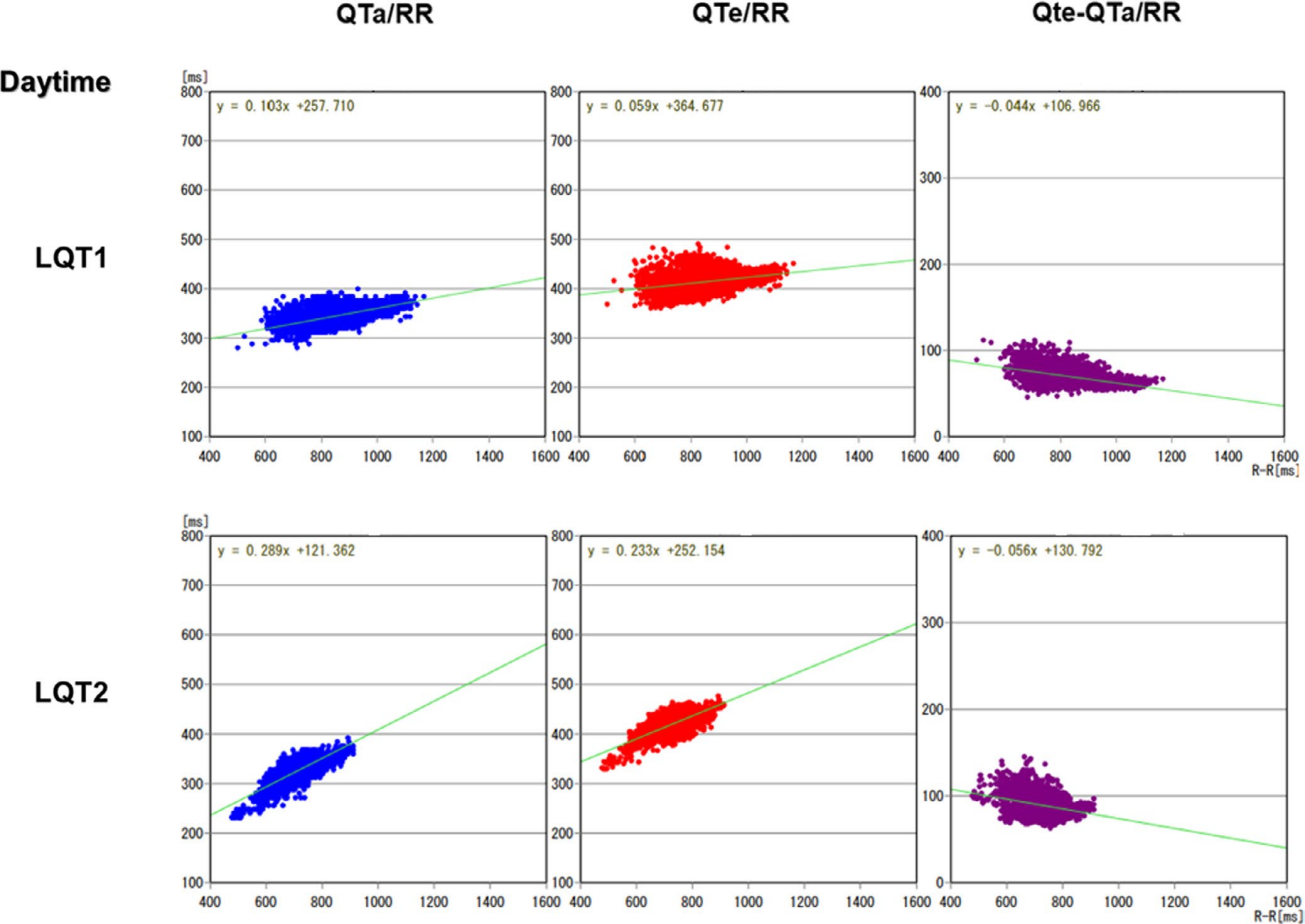
Representative QTa/RR, QTe/RR, and QTe‐QTa/RR slopes from daytime Holter recordings in the LQT1 and LQT2 patients. QTa/RR and QTe/RR slopes were steeper in the LQT2 patient than that of the LQT1 patient

**FIGURE 5:**
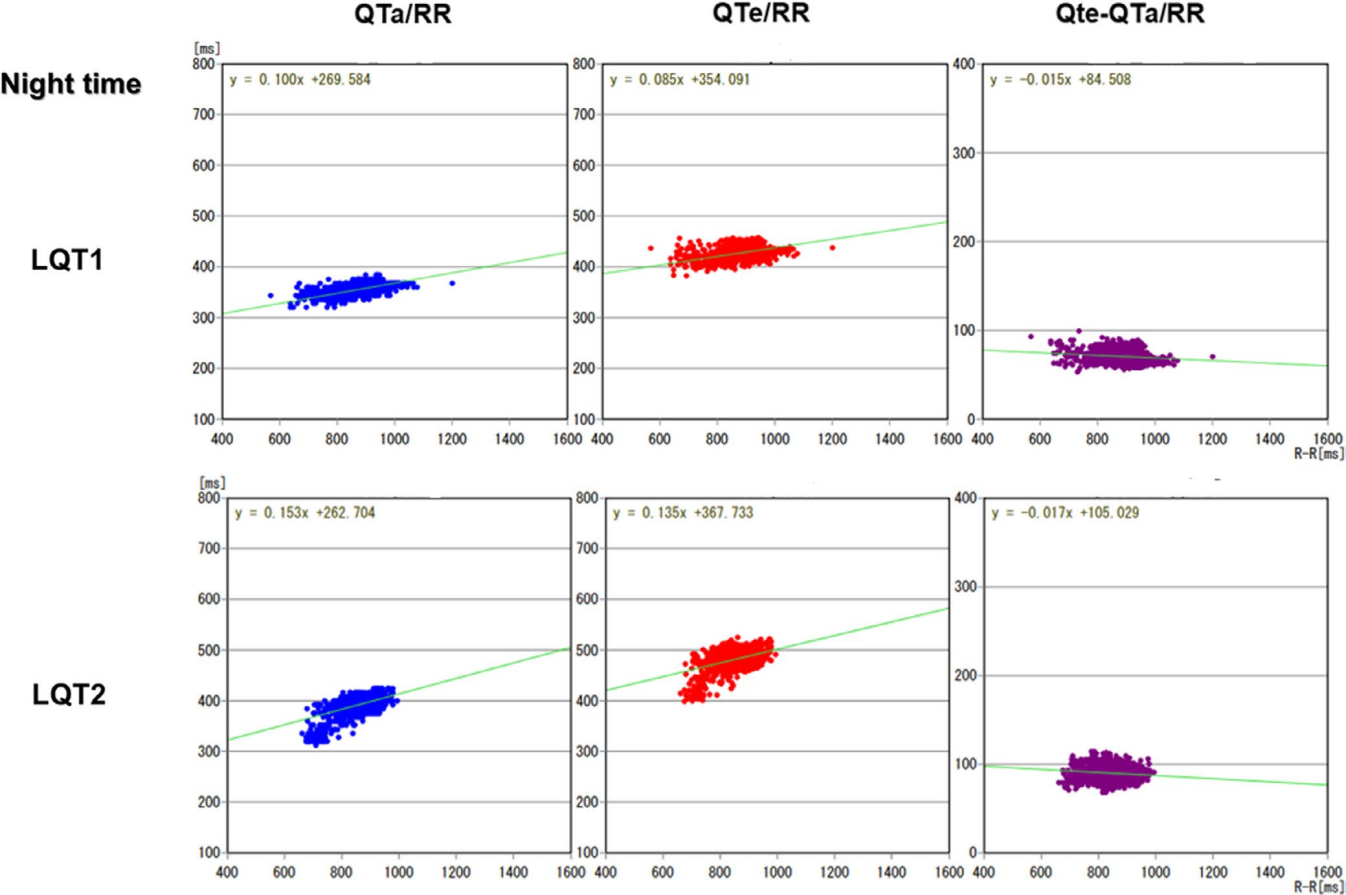
Representative QTa/RR, QTe/RR, and QTe‐QTa/RR slopes from night‐time Holter recordings in the LQT1 patients and LQT2 patients. Although QTa/RR slope was steeper in the LQT2 patient than that of the LQT1 patient, the degree was lower than that from entire 24‐hour or daytime Holter recordings

Average QTe was significantly longer, and QTe/RR and QTa/RR slopes from entire 24‐hour Holter recordings were significantly steeper in the LQT2 patients than those in the LQT1 patients (472.0 ± 40.6 vs. 447.1 ± 44.8 ms, *p* = .037; 0.262 ± 0.063 vs. 0.204 ± 0.055, *p* = .0007; 0.233 ± 0.052 vs. 0.181 ± 0.040, *p* = .0002, respectively). Representative QT trend graph in both groups is shown in Figure 2, and representative QTa/RR slopes from entire 24‐hour, daytime and night‐time Holter recordings in both groups are shown in Figures 3, 4, 5, respectively. QTe/RR and QTa/RR slopes from daytime Holter recordings in the LQT2 patients were also significantly steeper than those in the LQT1 patients (0.197 ± 0.057 vs. 0.158 ± 0.066, *p* = .024; 0.190 ± 0.048 vs. 0.153 ± 0.050, *p* = .008, Table 1). There were no significant differences in the other parameters (Table 1).

### 3.3 ROC analysis

**FIGURE 6:**
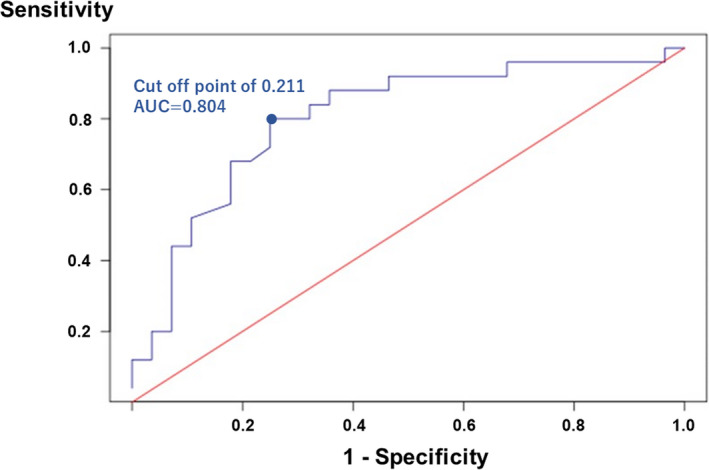
The receiver operating characteristic (ROC) curve analysis showed an optimal cutoff point of 0.211 of QTa/RR slope from entire 24‐hour Holter recordings, with 80.0% sensitivity, 75.0% specificity, and an area under the curve of 0.804 (95% confidence interval, 0.68–0.93)

The receiver operating curve analysis revealed that the QTa/RR slope of 0.211 at the entire 24‐hour Holter was the best cutoff value for differential diagnosis between LQT1 and LQT2 (sensitivity: 80.0%, specificity: 75.0%, and area under the curve: 0.804 [95% CI = 0.68–0.93], Figure 6). Meanwhile, it showed an optimal cutoff point of 0.255 of the QTe/RR slope with 60.0% sensitivity and 89.3% specificity. The area under the curve of 0.774 (95% confidence interval, 0.64–0.91) was lower than that of the QTa‐RR slope.

## 4 DISCUSSION

The present study demonstrated that QTe/RR and QTa/RR slopes from entire 24‐hour and daytime Holter recordings were significantly steeper in the LQT2 patients in contrary to LQT1 patients. A cutoff score of 0.211 of QTa/RR slope from entire 24‐hour Holter recordings was most optimal to differentiate LQT1 from LQT2 (sensitivity, 80%; specificity, 75%).

The identification of LQTS genotype is crucial because the treatment differs according to LQTS genotype. From an electrocardiographic point of view, broad‐based prolonged T waves are commonly observed in the LQT1 syndrome, whereas low‐amplitude T waves with a notched or bifurcated configuration are seen frequently in the LQT2 syndrome (Moss et al., 1995).

Zhang et al. have developed T‐wave patterns of LQT1 (infantile ST‐T wave, broad‐based, normal ‐appearing T wave, and late‐onset normal‐appearing T wave) and of LQT2 (obvious bifid T wave, subtle bifid T wave with second component on the top or the downslope, and low‐amplitude and widely split bifid T wave) to discriminate among LQTS patients with different genotypes. Using these patterns, cardiologists could identify LQT1 and LQT2 patients, but the sensitivity is not so high (61% and 62%, respectively) (Takenaka et al., 2003). Although the exercise‐stress test and epinephrine infusion test have been proposed for differential diagnosis between LQT1 and LQT2, they are provocative or invasive (Shimizu et al., 2004; Zhang et al., 2000). The findings of the present study suggest that QT/RR relationships may have additional value over standard 12‐lead ECG.

QT/RR relationships analyzed based on long‐term Holter recordings can evaluate the QT adaptation to a changing heart rate. It has been demonstrated that the QT/RR slope was significantly increased in patients with structural heart disease (Cygankiewicz et al., 2009; Iacoviello et al., 2007; Milliez et al., 2005). As for LQTS patients, a previous QT/RR relationship analysis showed a linear slope equal to 0.12 ± 0.04 in healthy subjects and a significantly higher slope in LQT1 and LQT2 carriers (QT slope >0.17). However, no significant difference was observed at the QT/RR slope between LQT1 and LQT2 (0.17 ± 0.10 vs. 0.22 ± 0.16) (Couderc et al., 2007). In contrast, Yamaguchi et al reported that QT/RR slope was significantly greater in LQT2 than in LQT1 patients (0.207 ± 0.032 vs. 0.163 ± 0.014, *p* < .05) (Yamaguchi et al., 2017).

In this multicenter study with all patients genetically identified, QTe/RR and QTa/RR slopes from entire 24‐hour and daytime Holter recordings were significantly steeper in the LQT2 patients compared to the LQT1 patients. Our findings support previous studies suggesting that QT/RR relationship may be useful for differential diagnosis between LQT1 and LQT2. The steeper QT/RR slope in the LQT2 than that in the LQT1 is at least due to more significant QT prolongation at an increased heart rate in the LQT1 compared to the LQT2 patients, resulting in a more gradual QT/RR slope at an increased heart rate in the LQT1 patients. Our result may support this speculation that the QT/RR slope at daytime, when a sympathetic tone is higher, was significantly steeper in the LQT2, whereas that at nighttime, when a sympathetic tone is lower, was not different between the LQT1 and LQT2. The QT/RR slope is influenced by autonomic balance and has circadian variations (Extramiana et al., 1999). Recently, Page et al. reported that LQT1 patients showed more frequent QTc prolongation during the day than night. In contrast, LQT2 patients showed less frequent QTc prolongation during the day than at night (Page et al., 2016).

QTe‐QTa is considered to reflect transmural dispersion of repolarization (TDR) and possibly useful for differential diagnosis between LQT1 and LQT2. Our previous study from the body surface potential mapping showed that the QTe‐QTa was more decreased in LQT1 than that in LQT2 patients after beta‐blockade administration (Shimizu et al., 2002). This may explain the reason why beta‐blockers are more effective in LQT1 than LQT2. In the present study, there were no significant differences in QTe‐QTa/RR slope between the LQT1 and the LQT2, although the reason of this finding is unclear.

### 4.1 Study limitations

Lack of control matched group is a major limitation of the study. Secondly, the sample size is relatively small and majority of patients were females, potentially resulting in selection bias. More extensive prospective studies are needed to confirm our findings and evaluate the QT/RR relationships’ clinical utility. However, our study has strength in that this is the first multicenter study, and all patients were genetically identified.

## 5 CONCLUSIONS

QT/RR relationships using 24‐hour Holter monitoring are feasible and may be useful for differential diagnosis between LQT1 and LQT2.

## CONFLICT OF INTEREST

K.Y., T.A., N.S., T.Y., H.M., Y.I., and Y.K. have no relationships to disclose. W.S. has received honoraria from Suzuken Co. Ltd.

## AUTHOR CONTRIBUTIONS

K.Y. contributed to data collection, data management and analyses, statistical analyses, and writing of the manuscript. W.S. contributed to data analyses, supervision, and revision of the manuscript. T.A., N.S., and Y.K. contributed to data collection, supervision, and revision of the manuscript. T.Y., H.M., and Y.I. contributed to supervision and revision of the manuscript.

## ETHICS

The study protocol was approved by the Institutional Review Board of each participating institution, and was conducted according to the principles of the Declaration of Helsinki. Written informed consent was obtained from all patients.

## Data Availability

The data that support the findings of this study are available from the corresponding author upon reasonable request.

## References

[anec12878-bib-0001] Couderc, J. P., Vaglio, M., Xia, X., McNitt, S., Wicker, P., Sarapa, N., Moss, A. J., & Zareba, W. (2007). Impaired T‐amplitude adaptation to heart rate characterizes I(Kr) inhibition in the congenital and acquired forms of the long QT syndrome. Journal of Cardiovascular Electrophysiology, 18, 1299–1305. 17916157 10.1111/j.1540-8167.2007.00960.x

[anec12878-bib-0002] Cygankiewicz, I., Zareba, W., Vazquez, R., Bayes‐Genis, A., Pascual, D., Macaya, C., Almendral, J., Fiol, M., Bardaji, A., Gonzalez‐Juanatey, J. R., Nieto, V., Valdes, M., Cinca, J., de Luna, A. B., & MUSIC Investigators . (2009). Risk stratification of mortality in patients with heart failure and left ventricular ejection fraction >35%. American Journal of Cardiology, 103, 1003–1010. 10.1016/j.amjcard.2008.11.061 19327431

[anec12878-bib-0003] Extramiana, F., Maison‐Blanche, P., Badilini, F., Pinoteau, J., Deseo, T., & Coumel, P. (1999). Circadian modulation of QT rate dependence in healthy volunteers: Gender and age differences. Journal of Electrocardiology, 32, 33–43. 10.1016/S0022-0736(99)90019-5. 10037087

[anec12878-bib-0004] Iacoviello, M., Forleo, C., Guida, P., Romito, R., Sorgente, A., Sorrentino, S., Catucci, S., Mastropasqua, F., & Pitzalis, M. (2007). Ventricular repolarization dynamicity provides independent prognostic information toward major arrhythmic events in patients with idiopathic dilated cardiomyopathy. Journal of the American College of Cardiology, 50, 225–231. 10.1016/j.jacc.2007.02.071 17631214

[anec12878-bib-0005] Merri, M., Moss, A. J., Benhorin, J., Locati, E. H., Alberti, M., & Badilini, F. (1992). Relation between ventricular repolarization duration and cardiac cycle length during 24‐hour Holter recordings. Findings in normal patients and patients with long QT syndrome. Circulation, 85, 1816–1821. 10.1161/01.CIR.85.5.1816 1572038

[anec12878-bib-0006] Milliez, P., Leenhardt, A., Maisonblanche, P., Vicaut, E., Badilini, F., Siliste, C., Benchetrit, C., Coumel, P., & EMIAT Investigators . (2005). Usefulness of ventricular repolarization dynamicity in patients with ischemic cardiomyopathy (from the European myocardial infarct amiodarone trial). American Journal of Cardiology, 95, 821–826. 10.1016/j.amjcard.2004.11.047 15781008

[anec12878-bib-0007] Moss, A. J., Shimizu, W., Wilde, A. A., Towbin, J. A., Zareba, W., Robinson, J. L., Qi, M., Vincent, G. M., Ackerman, M. J., Kaufman, E. S., Hofman, N., Seth, R., Kamakura, S., Miyamoto, Y., Goldenberg, I., Andrews, M. L., & McNitt, S. (2007). Clinical aspects of type‐1 long‐QT syndrome by location, coding type, and biophysical function of mutations involving the KCNQ1 gene. Circulation, 115, 2481–2489. 10.1161/CIRCULATIONAHA.106.665406. 17470695 PMC3332528

[anec12878-bib-0008] Moss, A. J., Zareba, W., Benhorin, J., Locati, E. H., Hall, W. J., Robinson, J. L., Schwartz, P. J., Towbin, J. A., Vincent, G. M., & Lehmann, M. H. (1995). ECG T‐wave patterns in genetically distinct forms of the hereditary long QT syndrome. Circulation, 92, 2929–2934. 10.1161/01.CIR.92.10.2929 7586261

[anec12878-bib-0009] Nemec, J., Buncová, M., Bůlková, V., Hejlik, J., Winter, B., Shen, W. K., & Ackerman, M. J. (2004). Heart rate dependence of the QT interval duration: Differences among congenital long QT syndrome subtypes. Journal of Cardiovascular Electrophysiology, 15, 550–556. 10.1046/j.1540-8167.2004.03096.x 15149424

[anec12878-bib-0010] Neyroud, N., Maison‐Blanche, P., Denjoy, I., Chevret, S., Donger, C., Dausse, E., Fayn, J., Badilini, F., Menhabi, N., Schwartz, K., Guicheney, P., & Coumel, P. (1998). Diagnostic performance of QT interval variables from 24‐h electrocardiography in the long QT syndrome. European Heart Journal, 19, 158–165. 10.1053/euhj.1997.0730 9503190

[anec12878-bib-0011] Page, A., Aktas, M. K., Soyata, T., Zareba, W., & Couderc, J. P. (2016). "QT clock" to improve detection of QT prolongation in long QT syndrome patients. Heart Rhythm, 13, 190–198. 10.1016/j.hrthm.2015.08.037 26334569 PMC4698188

[anec12878-bib-0012] Schwartz, P. J., Periti, M., & Malliani, A. (1975). The long QT syndrome. American Heart Journal, 89, 378–390. 10.1016/0002-8703(75)90089-7 234667

[anec12878-bib-0013] Shimizu, W. (2005). The long QT syndrome: Therapeutic implications of a genetic diagnosis. Cardiovascular Research, 67, 347–356. 10.1016/j.cardiores.2005.03.020 15979599

[anec12878-bib-0014] Shimizu, W., Moss, A. J., Wilde, A. A., Towbin, J. A., Ackerman, M. J., January, C. T., Tester, D. J., Zareba, W., Robinson, J. L., Qi, M., Vincent, G. M., Kaufman, E. S., Hofman, N., Noda, T., Kamakura, S., Miyamoto, Y., Shah, S., Amin, V., Goldenberg, I., … McNitt, S. (2009). Genotype‐phenotype aspects of type‐2 long‐QT syndrome. Journal of the American College of Cardiology, 54, 2052–2062. 10.1016/j.jacc.2009.08.028 19926013 PMC2808400

[anec12878-bib-0015] Shimizu, W., Noda, T., Takaki, H., Nagaya, N., Satomi, K., Kurita, T., Suyama, K., Aihara, N., Sunagawa, K., Echigo, S., Miyamoto, Y., Yoshimasa, Y., Nakamura, K., Ohe, T., Towbin, J. A., Priori, S. G., & Kamakura, S. (2004). Diagnostic value of epinephrine test for genotyping LQT1, LQT2, and LQT3 forms of congenital long QT syndrome. Heart Rhythm, 1, 276–283. 10.1016/j.hrthm.2004.04.021 15851169

[anec12878-bib-0016] Shimizu, W., Tanabe, Y., Aiba, T., Inagaki, M., Kurita, T., Suyama, K., Nagaya, N., Taguchi, A., Aihara, N., Sunagawa, K., Nakamura, K., Ohe, T., Towbin, J. A., Priori, S. G., & Kamakura, S. (2002). Differential effects of beta‐blockade on dispersion of repolarization in the absence and presence of sympathetic stimulation between the LQT1 and LQT2 forms of congenital long QT syndrome. Journal of the American College of Cardiology, 39, 1984–1991. 10.1016/S0735-1097(02)01894-6 12084597

[anec12878-bib-0017] Takenaka, K., Ai, T., Shimizu, W., Kobori, A., Ninomiya, T., Otani, H., Kubota, T., Takaki, H., Kamakura, S., & Horie, M. (2003). Exercise stress test amplifies genotype‐phenotype correlation in the LQT1 and LQT2 forms of the long‐QT syndrome. Circulation, 107, 838–844. 10.1161/01.CIR.0000048142.85076.A2 12591753

[anec12878-bib-0018] Viitasalo, M., Oikarinen, L., Väänänen, H., Swan, H., Piippo, K., Kontula, K., Barron, H. V., Toivonen, L., & Scheinman, M. M. (2002). Differentiation between LQT1 and LQT2 patients and unaffected subjects using 24‐hour electrocardiographic recordings. American Journal of Cardiology, 89, 679–685. 10.1016/S0002-9149(01)02339-6 11897209

[anec12878-bib-0019] Yamaguchi, Y., Mizumaki, K., Hata, Y., Sakamoto, T., Nakatani, Y., Kataoka, N., Ichida, F., Inoue, H., & Nishida, N. (2017). Latent pathogenicity of the G38S polymorphism of KCNE1 K+ channel modulator. Heart and Vessels, 32, 186–192. 27255646 10.1007/s00380-016-0859-1

[anec12878-bib-0020] Zhang, L., Timothy, K. W., Vincent, G. M., Lehmann, M. H., Fox, J., Giuli, L. C., Shen, J., Splawski, I., Priori, S. G., Compton, S. J., Yanowitz, F., Benhorin, J., Moss, A. J., Schwartz, P. J., Robinson, J. L., Wang, Q., Zareba, W., Keating, M. T., Towbin, J. A., … Medina, A. (2000). Spectrum of ST‐T‐wave patterns and repolarization parameters in congenital long‐QT syndrome: ECG findings identify genotypes. Circulation, 102, 2849–2855. 10.1161/01.CIR.102.23.2849 11104743

